# Supplementary material for: Diversity of a Lactic Acid Bacterial Community during Fermentation of Gajami-Sikhae, a Traditional Korean Fermented Fish, as Determined by Matrix-Assisted Laser Desorption/Ionization Time-of-Flight Mass Spectrometry
Source: Foods. 2022 Mar 22;11(7):909. doi: 10.3390/foods11070909 (PMC8997922; doi:10.3390/foods11070909)
Supplement: Supplementary file 1 [file foods-11-00909-s001.zip › foods-1626144-supplementary.pdf]

Table S1. Information on sample type (fermented temperature and fermented period).

Sample no.	Temperature (°C)	Period (days)
C	-	0
A5	5°C	5 days
A10	5°C	10 days
A15	5°C	15 days
A20	5°C	20 days
A25	5°C	25 days
A30	5°C	30 days
A40	5°C	40 days
A60	5°C	60 days
B5	10°C	5 days
B10	10°C	10 days
B15	10°C	15 days
B20	10°C	20 days
B25	10°C	25 days
B30	10°C	30 days
B40	10°C	40 days
B60	10°C	60 days
C1	15°C	1 day
C3	15°C	3 days
C5	15°C	5 days
C7	15°C	7 days
C10	15°C	10 days
C15	15°C	15 days
C20	15°C	20 days
C25	15°C	25 days
C30	15°C	30 days
C40	15°C	40 days
C60	15°C	60 days
D1	20°C	1 day
D3	20°C	3 days
D5	20°C	5 days
D7	20°C	7 days
D10	20°C	10 days
D15	20°C	15 days
D20	20°C	20 days
D25	20°C	25 days
D30	20°C	30 days
D40	20°C	40 days
D60	20°C	60 days

Table S2. Difference in pH and acidity values according to the fermentation period.

No.	Temperature (5°C)		No.	Temperature (10°C)		No.	Temperature (15°C)		No.	Temperature (20°C)	
	pH	Acidity (%)		pH	Acidity (%)		pH	Acidity (%)		pH	Acidity (%)
C	6.25 ± 0.01 a	0.33 ± 0.03 e	C	6.25 ± 0.01 a	0.33 ± 0.03 f	C	6.25 ± 0.01 a	0.33 ± 0.03 i	C	6.25 ± 0.01 a	0.33 ± 0.03 k
-	-	-	-	-	-	C1	6.18 ± 0.01 b	0.42 ± 0.03 h	D1	4.61 ± 0.01 b	1.00 ± 0.05 j
-	-	-	-	-	-	C3	4.81 ± 0.01 c	0.84 ± 0.02 g	D3	4.50 ± 0.01 c	1.10 ± 0.02 i
A5	6.06 ± 0.01 b	0.37 ± 0.02 e	B5	4.73 ± 0.01 b	0.95 ± 0.02 e	C5	4.49 ± 0.01 d	1.11 ± 0.01 f	D5	4.44 ± 0.01 d	1.28 ± 0.01 h
-	-	-	-	-	-	C7	4.46 ± 0.01 e	1.19 ± 0.02 e	D7	4.39 ± 0.01 d	1.39 ± 0.04 g

A10	4.81 ± 0.01 c	0.80 ± 0.04 d	B10	4.45 ± 0.00 c	1.21 ± 0.02 d	C10	4.37 ± 0.00 f	1.42 ± 0.07 d	D10	4.26 ± 0.01 e	1.60 ± 0.04 f
A15	4.54 ± 0.01 d	1.09 ± 0.03 c	B15	4.44 ± 0.01 c	1.26 ± 0.03 c	C15	4.26 ± 0.00 g	1.62 ± 0.03 c	D15	4.09 ± 0.01 f	1.88 ± 0.02 e
A20	4.49 ± 0.01 e	1.18 ± 0.02 b	B20	4.40 ± 0.00 d	1.32 ± 0.01 b	C20	4.27 ± 0.02 rh	1.60 ± 0.02 c	D20	4.08 ± 0.01 f	1.98 ± 0.01 d
A25	4.48 ± 0.00 f	1.16 ± 0.02 b	B25	4.38 ± 0.03 d	1.32 ± 0.03 b	C25	4.24 ± 0.02 hi	1.62 ± 0.03 c	D25	3.98 ± 0.01 g	2.48 ± 0.02 c
A30	4.48 ± 0.02 f	1.17 ± 0.01 b	B30	4.33 ± 0.03 e	1.49 ± 0.02 a	C30	4.22 ± 0.03 i	1.66 ± 0.04 bc	D30	3.98 ± 0.00 g	2.50 ± 0.05 c
A40	4.46 ± 0.00 g	1.18 ± 0.02 b	B40	4.34 ± 0.01 e	1.50 ± 0.03 a	C40	4.20 ± 0.01 j	1.70 ± 0.02 b	D40	3.90 ± 0.09 h	2.58 ± 0.03 b
A50	4.46 ± 0.01 g	1.23 ± 0.03 a	B50	4.34 ± 0.01 e	1.52 ± 0.03 a	C50	4.12 ± 0.02 k	1.86 ± 0.03 a	D50	3.84 ± 0.01 i	2.77 ± 0.02 a
A60	4.45 ± 0.01 g	1.26 ± 0.03 a	B60	4.33 ± 0.02 e	1.53 ± 0.03 a	C60	4.10 ± 0.02 k	1.84 ± 0.04 a	D60	3.80 ± 0.06 i	2.78 ± 0.02 a

Data values indicated as the mean ± standard deviation of three replications. Values flowed by different letters in fermentation period are significantly different ($p < 0.05$).

Table S3. Number of strains isolated from gajami-sikhae at the genus level.

Genus	No. of isolates
Bacillus	2
Enterobacter	1
Enterococcus	30
Lactiplantibacillus	661
Lactobacillus	1
Latilactobacillus	2182
Levilactobacillus	643
Lactococcus	7
Leuconostoc	622
Pediococcus	8
Weissella	667

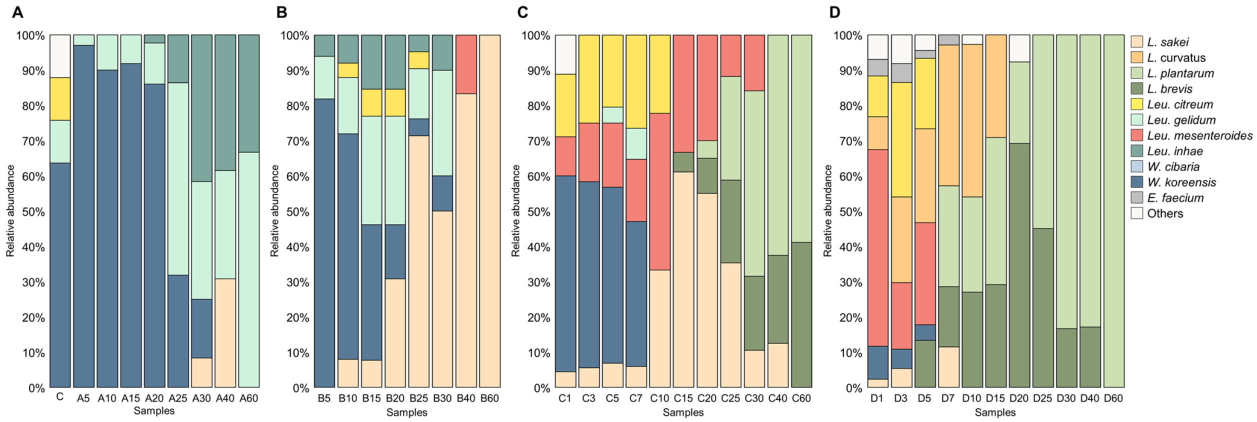

Figure S1. Changes in the LAB communities during the fermentation at (A) 5°C, (B) 10°C, (C) 15°C, and (D) 20°C. A graph generated by considering only for the 1019 cultures identified at the genus level (1.700–1.999). The others indicate species with a prevalence of 0.15%, including *P. pentosaceus*, *Lc. lactis*, *W. viridescens*, *W. hellenica*, *Leu. lactis*, and *W. kandleri*.
